# Spontaneous Magnetic Alignment by Yearling Snapping Turtles: Rapid Association of Radio Frequency Dependent Pattern of Magnetic Input with Novel Surroundings

**DOI:** 10.1371/journal.pone.0124728

**Published:** 2015-05-15

**Authors:** Lukas Landler, Michael S. Painter, Paul W. Youmans, William A. Hopkins, John B. Phillips

**Affiliations:** 1 Department of Biological Sciences, Virginia Tech, Blacksburg, Virginia, United States of America; 2 Department of Fish and Wildlife Conservation, Virginia Tech, Blacksburg, Virginia, United States of America; Bowling Green State Universtiy, UNITED STATES

## Abstract

We investigated spontaneous magnetic alignment (SMA) by juvenile snapping turtles using exposure to low-level radio frequency (RF) fields at the Larmor frequency to help characterize the underlying sensory mechanism. Turtles, first introduced to the testing environment without the presence of RF aligned consistently towards magnetic north when subsequent magnetic testing conditions were also free of RF (‘RF off → RF off’), but were disoriented when subsequently exposed to RF (‘RF off → RF on’). In contrast, animals initially introduced to the testing environment with RF present were disoriented when tested without RF (‘RF on → RF off’), but aligned towards magnetic south when tested with RF (‘RF on → RF on’). Sensitivity of the SMA response of yearling turtles to RF is consistent with the involvement of a radical pair mechanism. Furthermore, the effect of RF appears to result from a change in the pattern of magnetic input, rather than elimination of magnetic input altogether, as proposed to explain similar effects in other systems/organisms. The findings show that turtles first exposed to a novel environment form a lasting association between the pattern of magnetic input and their surroundings. However, under natural conditions turtles would never experience a change in the pattern of magnetic input. Therefore, if turtles form a similar association of magnetic cues with the surroundings each time they encounter unfamiliar habitat, as seems likely, the same pattern of magnetic input would be associated with multiple sites/localities. This would be expected from a sensory input that functions as a global reference frame, helping to place multiple locales (i.e., multiple local landmark arrays) into register to form a global map of familiar space.

## Introduction

Turtles are among the wide variety of animals known to be sensitive to the Earth’s magnetic field [1–4]. However, the nature of the underlying magnetoreception mechanism has yet to be determined [5–7]. In terrestrial animals, there is evidence for two distinct mechanisms of magnetoreception [6, 7]; the magnetite-based mechanism (MBM) involving single domain or super-paramagnetic particles of biogenic magnetite [6, 8, 9], and the radical pair mechanism (RPM) involving a light-dependent biochemical reaction that forms long-lived, spin-coherent radical pairs. A specialized class of photopigments (i.e., cryptochromes) has been suggested to play a central role in the RPM [10–13] with the alignment of the magnetic field modulating the response of these photopigments to light [14]. Importantly, these two mechanisms are not mutually exclusive. For example, in amphibians and birds a MBM is thought to derive geographic position (“map”) information, while the light-dependent RPM provides directional (“compass”) information ([15], but see [16, 17] for MBM based compass orientation in subterranean mammals). The use of a light-dependent magnetic compass, despite the presence of a magnetite-based receptor with properties arguably better suited for this task (i.e. sensitivity to the polarity of the magnetic field, ability to operate in total darkness), suggests that the light-dependent mechanism may provide more than simple directional information [18].

Behavioral studies have helped characterize the functional properties of both the MBM and the RPM [5, 15, 19–26]. For example, in insects, amphibians and birds, the magnetic compass has been shown to be dependent on light [23, 24, 27–31], and sensitive to the inclination, but not polarity, of the magnetic field [22, 32]. Both results are consistent with a RPM [33–35]. In contrast, mole-rats that inhabit aphotic subterranean habitats have a MBM-based magnetic compass that operates in total darkness and is sensitive to the polarity of the magnetic field [16]. To date, responses showing properties of both mechanisms have only been observed in animals that use magnetic cues in both the map and compass components of long-distance navigation (newts [22, 36], birds [37, 38] and sea turtles [39]), suggesting that these responses may receive inputs from both mechanisms, rather than the involvement of a third, distinct mechanism (see below).

A diagnostic property that distinguishes unambiguously between a MBM and RPM is sensitivity of the RPM to low-level radio frequency (RF) fields [40]. For instance, magnetic compass orientation of birds is disrupted by low-level RF fields at the Larmor frequency (i.e., the precession frequency of an electron spin in the ambient magnetic field) [41], as well as low-level RF noise, consistent with the involvement of a RPM [40]. Recent findings suggest that the threshold of sensitivity of a RPM based system may be as low as 1 nT (RF intensity), which can be in the range of anthropogenic electromagnetic noise [42]. In contrast, non-light-dependent, SMA of mole-rats was unaffected by RF fields at intensities as high as 4800 nT [43], as would be expected for a MBM.

Interest in SMA, first reported in several insects [44], is experiencing a revival in the current literature [45–50]. Spontaneous magnetic alignment is defined here as an untrained, non-goal-directed, body alignment relative to the Earth´s magnetic field (often resting or landing positions). Although the proximate and ultimate causes of SMA are not understood, it may serve a variety of important functions. For example, it has been suggested that maintaining a fixed alignment relative to the magnetic north-south axis may facilitate measurement of magnetic inclination and/or total intensity, increase the accuracy of 3-dimensional (3-D) targeting and course control, and/or help to encode spatial input from other sensory modalities [18, 46, 51, 52].

The putative magneto-receptive molecule for a RPM, cryptochrome [53, 54], has been shown to occur in photoreceptors in the pineal complex and/or retina of vertebrates, and compound eye of insects [35, 55–57]. Specific classes of photoreceptors may be specialized for sensing the geomagnetic field, e.g. the UV/violet cones in the retina of birds, extra-ocular photoreceptors mediating chromatic responses in the pineal complex of amphibians [35], and central retinula cells in the compound eyes of flies [7]. In animals, in which the specialized photoreceptors are located in the retina or compound eye, the magnetic field may be perceived as a 3-dimensional ‘visual’ pattern that appears to surround the animal and be superimposed on its surroundings ([11, 14, 18, 58–60]; and see below). In effect, the animal would be at the center of a simple spherical coordinate system that is fixed in alignment relative to the magnetic field as it moves through the environment.

In the present study, experiments were carried out to determine if an earth-strength magnetic field influences alignment behavior of yearling snapping turtles (*Chelydra serpentina*). In particular, the experiments focused on the role that magnetic cues might play when an animal is introduced to a novel environment. Exposure to a low-level RF field was used to determine whether a RPM was involved in the SMA of turtles, and whether the conditions under which the turtles were first introduced to the testing apparatus (with or without low-level RF fields) influenced subsequent alignment responses to the magnetic field.

## Results

The alignments of turtles tested in a vertical magnetic field, which does not provide directional information, were randomly distributed (S1 Fig) indicating that no other non-magnetic (e.g. topographic) directional cues were used by the animals in the testing arena.

Testing turtles in four symmetrical horizontal magnetic field alignments (magnetic N = topographic N, E, S, W) made it possible to partition the alignment responses into topographic and magnetic components (see Material and Methods). In all treatment conditions, topographic distributions were indistinguishable from random (S2 Fig), providing further evidence that the animals were not relying on non-magnetic cues.

**Fig 1:**
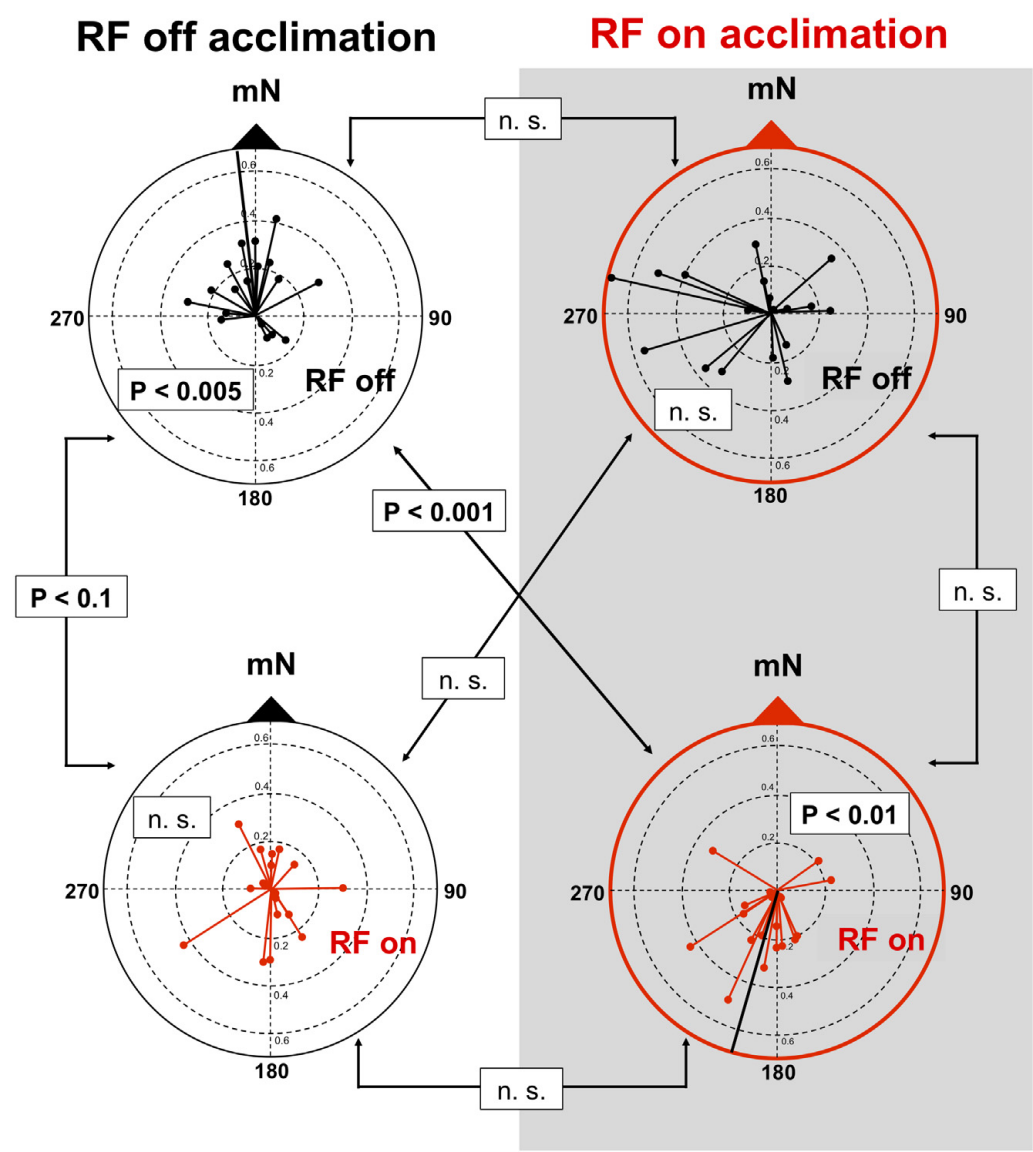
Magnetic component of responses of individual turtles plotted relative to magnetic North (mN). Magnetic directional preferences of turtles combined from four magnetic field alignments (magnetic north aligned in each of the four cardinal directions). Responses of turtles initially exposed to the magnetic field without RF are shown in the left column with black outer circles. Responses of turtles initially exposed to the magnetic field in the presence of the RF stimulus are shown in the right column with red outer circles. Black lines connected to black dots (labeled “RF off”) are the mean vectors showing the magnetic component pooled from the four magnetic field alignments in which the turtles were tested without RF, and the responses shown in red (labeled “RF on”) are from the four magnetic fields alignments in which the same turtles were tested in the presence of RF. Moore’s modified Rayleigh test was used to test each distribution for non-random unimodal alignment. Dependent data, e.g., diagrams in the left column or in the right column with the same colored circles (showing data collected from the same individuals), were tested for significant differences using Moore’s paired sample test. Independent data (i.e. distributions in the same horizontal row obtained from different individuals) were tested for significant differences using the Mardia’s two-sample test.

Animals initially exposed to the geomagnetic field without RF and subsequently tested in the four magnetic field alignments without RF (‘RF off → RF off’ condition) showed consistent alignment relative to the magnetic field with the mean vector bearing towards magnetic north (mean direction: 353°, p < 0.005, R* = 1.388, n = 18, Moore’s modified Rayleigh test, Fig 1). When tested in the four magnetic field alignment with RF (‘RF off → RF on’ condition) these same animals failed to exhibit a consistent alignment relative to the magnetic field (n. s., R* = 0.121, n = 18, Moore’s modified Rayleigh test). The difference between the ‘RF off → RF on’ and ‘RF off → RF off’ responses approached significance (p < 0.1, R' = 1.005, n = 18, Moore’s paired two-sample test).

The behavior of animals initially exposed to the geomagnetic field with RF present was remarkably different. After initial exposure to the magnetic field with RF present, animals subsequently tested in the four magnetic field alignments without RF (RF on → RF off; Fig 1) were disoriented (n. s., R* = 0.674, n = 18, Moore’s modified Rayleigh test). However, in the four magnetic field alignments with RF present (RF on → RF on), the same animals showed consistent alignment relative to the magnetic field (mean direction: 196°, p < 0.01, R* = 1.307, n = 18, Moore’s modified Rayleigh test). Interestingly, however, the direction of magnetic alignment was toward magnetic south, i.e., opposite that of the ‘RF off → RF off’ group (Fig 1), and the distribution of magnetic responses in the two groups of turtles that were pre-exposed and tested in the same RF condition (‘RF off → RF off’, and ‘RF on → RF on’) were significantly different (p < 0.001, U = 0.42, n = 18, Mardia’s two-sample test, Fig 1).

## Discussion

Spontaneous magnetic preferences are known for many different taxa, from arthropods to mammals [50]. In vertebrates, these behaviors typically consist of unimodal or axial responses aligned near the magnetic north-south axis [50]. In the present experiments, directional responses of snapping turtles were tested in four symmetrical magnetic field alignments, which made it possible to isolate the component of the behavior that showed a consistent alignment relative to the magnetic field (see Methods). In the absence of RF exposure (‘RF off → RF off’ condition), the turtles’ directional responses showed a consistent northward alignment relative to the magnetic field, providing the first evidence for SMA in a reptile.

Previous experiments with migratory birds have shown that exposure to a RF field at the Larmor frequency at intensities as low as 15 nT can abolish magnetic compass orientation [61]. In the present experiments, turtles that were initially exposed to the testing apparatus without RF, failed to exhibit a consistent alignment relative to the magnetic field when subsequently tested in four symmetrical magnetic field alignments with RF present (‘RF off → RF on’ condition). Because the energy of interaction of the radio frequency field used in the present experiments (1.43 MHz at 30–52 nT) with an unpaired electron is many orders of magnitude below the thermal noise floor [40], this result is compelling evidence for a quantum process, such as the RPM, underlying the turtles’ SMA responses.

In contrast to turtles introduced to the testing chamber without RF and tested with RF (‘RF off → RF on’ condition), turtles introduced to the testing chamber in the presence of RF showed consistent alignment relative to the four magnetic field alignments when RF was present (‘RF on → RF’ on condition). However, they were disoriented when tested in the same four field alignments without RF (‘RF on → RF off’ condition). Moreover, the directional response of turtles in the ‘RF on → RF on’ condition (towards magnetic south) was opposite to that of turtles in the ‘RF off → RF off’ condition (towards magnetic north). Taken together, these findings indicate that RF fundamentally alters, but does not abolish, the directional information that the turtles obtained from the magnetic field. These findings also show that the presence or absence of RF when an animal is first introduced to novel surroundings influences its subsequent response to the magnetic field, i.e., turtles only showed a consistent alignment relative to the magnetic field in the same RF condition in which they were first introduced to the testing apparatus.

Our findings are consistent with evidence from animals as different as flies and mice that a complex, 3-D pattern is generated by the RPM [34]. In studies in which animals are trained to orient in a particular heading relative to the magnetic field [24, 51, 62, 63], and/or have been shown to calibrate the magnetic compass relative to a global reference system (e.g. [64]), failure to ‘recognize’ an altered pattern of magnetic input might have prevented the animals from orienting in an RF condition that differed from the one in which they had learned, or calibrated, the compass response [11]. However, because yearling snapping turtles in the present study exhibited spontaneous (i.e., unlearned and uncalibrated) alignment relative to the magnetic field (Fig 1), neither of these explanations can account for the dependence of the effects of RF exposure on initial experience in the novel testing environment.

Instead, the findings suggest that yearling turtles formed a rapid association between the novel surroundings and RPM response pattern, which differed depending on the initial RF exposure. This apparently spontaneous association seems to have a ‘sensitive period’ when the turtle is first introduced to novel surroundings during which this association is formed. The association lasted for a minimum of several hours (the duration of testing in the present experiment), and was unaffected by subsequent exposure to a pattern of magnetic input that differed depending on the RF-exposure condition. Further experiments are needed to determine whether the association persists over longer periods of time and is retained when an individual is removed and subsequently returned to the surroundings.

Under natural conditions turtles would never experience a change in the pattern of magnetic input. Therefore, if turtles form a similar association of magnetic cues with the surroundings each time they encounter unfamiliar habitat, as seems likely, the same pattern of magnetic input would be associated with multiple sites/localities. This would be expected from a sensory input that functions as a global reference frame, helping to place multiple locales (i.e., multiple local landmark arrays) into register to form a global map of familiar space.

The turtles response to magnetic cues in the presented experiments parallels the earlier findings of Collett and Baron (65] who showed that honeybees revisiting a familiar environment align themselves relative to the magnetic field so that they faced a radially-symmetrical visual landmark from a consistent magnetic direction. Based on these findings, as well as their earlier research [66, 67], the authors suggested that magnetic input provides honeybees with a coordinate system that simplifies spatial pattern recognition by aligning the animals so that “the pattern of landmarks imaged on their retina matches the pattern stored on previous visits to that place”[65]. Perhaps, as suggested by [18], the pattern of magnetic input from the RPM is ‘mapped’ onto the local environment analogous to taking a mental ‘snap shot’ of the novel surroundings with the 3-D ‘grid’ superimposed. Although much remains to be learned about the role of the magnetic field in encoding spatial information in vertebrates, evidence that mice require only two brief (< 60 sec) trials to encode the magnetic direction of a submerged platform in a ‘plus’ water maze task [51] clearly indicates the need for further investigation of the role of magnetic cues in organizing spatial information in novel surroundings.

Given the likelihood that exposure to low-level RF fields directly affects the RPM [40, 61], the findings reported here place important constraints on the properties of the underlying biophysical mechanism (Peter Hore, personal communication). The ‘reference-probe’ radical pair design proposed by Ritz, Wiltschko [68], in which one member of the radical pair is devoid of hyperfine interactions, would predict a strong resonant response to the Larmor frequency (used in this study) that would reduce the anisotropy of the response of the radical pair and thereby destroy the directional information required for magnetic orientation [69]. However, our results suggest that RF at the Larmor frequency is modifying the response of the RPM, altering rather than eliminating the resulting directional information. Such an effect could arise from a more complex radical pair design in which both radicals have hyperfine interactions. For instance, FAD-tryptophan could be molecules involved in such a mechanism (as originally proposed by Ritz, Adem (14]). Importantly, however, in order for RF fields at such low intensities to affect the radical pair, the spin-relaxation would need to be very slow, on the order of several milliseconds, which is unprecedented in known radical pair systems (Peter Hore, personal communication). Moreover, a FAD-tryptophan radical pair would be expected to exhibit peaks of sensitivity to RF fields outside the Larmor frequency range, as may be the case in migratory birds [42, 61].

In conclusion, the present study demonstrates spontaneous magnetic alignment by a reptile similar to that reported previously in other vertebrates, and shows the sensitivity of SMA to low-level RF fields at the Larmor Frequency. On the one hand, the findings confirm how little is actually known about the biophysical process underlying the RF-dependent magnetoreception mechanism, despite evidence that this type of mechanism may be present in animals as diverse as birds [61], epigeic rodents [34], reptiles (this study), amphibians [23], and some insects [70]. What is arguably the most important finding from these experiments, however, is that in addition to the well-studied use in goal-directed orientation, the magnetic field appears to play an important, and as yet poorly understood role, in encoding spatial information in the animal’s immediate surroundings.

## Materials and Methods

### Turtle collection and husbandry

From April-July 2011, eggs were collected and incubated from gravid female *C*. *serpentina* captured along the South River and from nearby sites along the Middle River (both Virginia, USA) using baited hoop traps (see [71, 72] for more information on trapping, egg collection and incubation, see also S2 Table). Once the incubated eggs hatched in Aug-Sept, individual hatchlings were housed in plastic, opaque Ziploc containers (591 ml; S.C. Johnson & Son, LLC, Wisconsin, USA) filled with 150 ml dechlorinated water placed in an environmental chamber at 25°C (observed temperature 25.46 ± 0.01°C) with a 12:12 light:dark cycle. Beginning in Oct, hibernation was induced by gradually (1°C increments) reducing temperature each week to 4.5°C. After 5 months in hibernation, temperatures were gradually increased to 25°C. The turtles were tested within one month of emergence in May 2012. All hatchlings were released at the end of the study at the site of maternal origin.

### Testing procedure

Testing trials took place in a double-walled testing building to minimize external sources of variation, such as vibration and sound cues. Additionally, the testing apparatus was located in a grounded faraday cage, and all power lines going into the enclosure were equipped with EMI/RF filters (Dearborn, 1JX2459) to reduce background electromagnetic noise. The testing apparatus (including the testing arena and surrounding magnetic coils; see below) was mounted on a ~400 kg sandbox supported by a ~10 cm layer of compressed fiberglass insulation to further reduce substrate vibrations.

**Fig 2:**
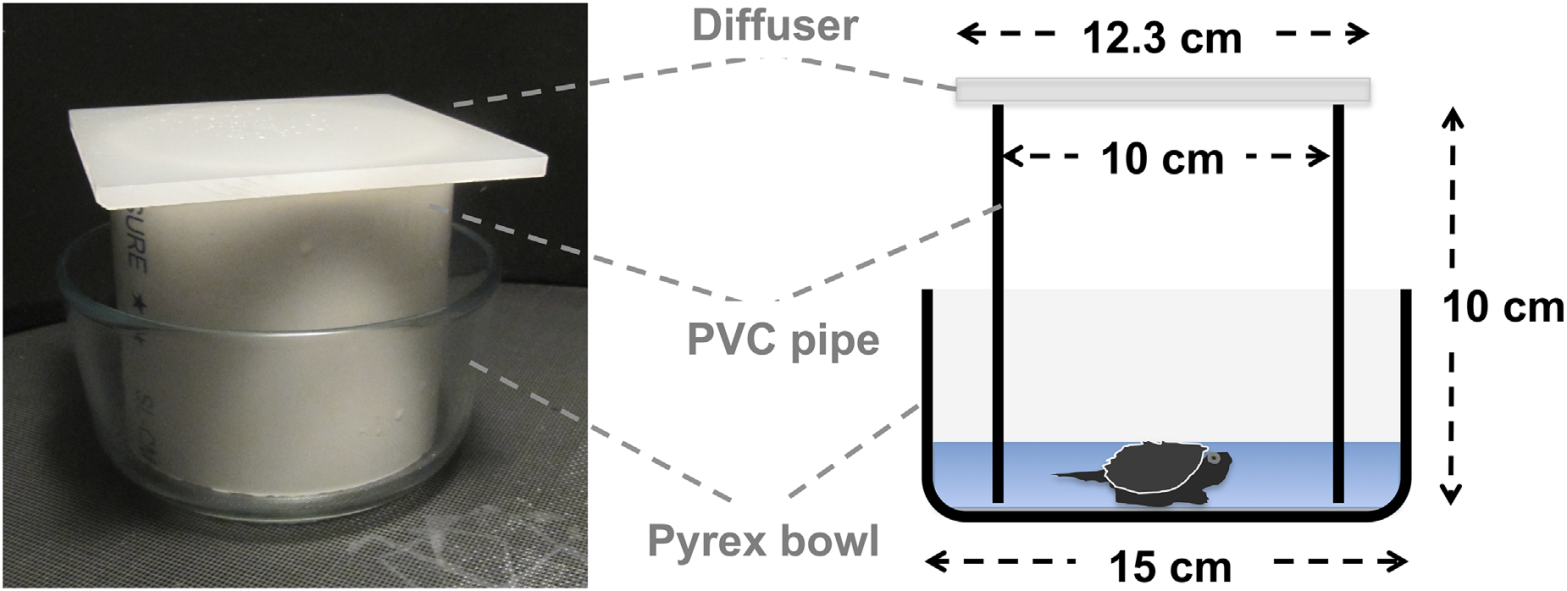
The experimental chamber. Turtles were tested in a Pyrex bowl with water that was ~1 cm deep so the turtle’s shell was not completely submerged. The Pyrex bowl, PVC surround, and overhead diffuser provided uniform visual surroundings.

In each testing session, six yearling snapping turtles were placed in individual testing chambers (Fig 2) inside a testing apparatus surrounded by a double-wrapped, cube surface coil [73]. Full-spectrum light was provided by an overhead light source centered above the testing arena, but outside the magnetic coils. The light passed through two frosted Pyrex glass diffusers before reaching the testing arena. Turtles were tested inside individual testing chambers consisting of a Pyrex bowl, a PVC cylinder inside the bowl that visually isolated each subject, and a milky white Plexiglas diffuser placed on top of each testing chamber (Fig 2). The chambers were placed on the floor of the testing apparatus made from a clear glass plate covered by gray fiberglass insect screen (light reflection from the screen prevented the animals from seeing through the bottom of the glass bowls and, therefore, from responding to any visual asymmetries beneath the arena).

Each test day, all 6 turtles were exposed to nine consecutive treatments that were determined in a pseudorandom order. Turtles exposed to RF in the first of the 9 testing conditions (3 of the 6 testing days) were exposed to RF during the 20 min acclimation period prior to the start of testing. Experimental treatments were as follows: vertical field only (no directional magnetic cues); mN = topographic north, south, east and west with RF present; mN = topographic north, south, east, and west with RF absent. Each individual was exposed to each of the nine treatments once for at least one hour. One hour of video was recorded in each condition, and only the last 40 minutes were used for the body alignment analysis. Animals were not removed from the testing chambers or handled between treatments; however, there was always at least a 20-minute acclimation period in each new treatment condition, before observations of body alignment were recorded.

All changes in magnetic field conditions and RF conditions were produced from outside the Faraday cage. Other than the electromagnetic changes, the turtles were not exposed to disturbance of any kind nor did the experimenter enter the Faraday cage that surrounded the testing apparatus during the entire sequence of treatment conditions.

The behavior of the turtles was recorded from underneath the testing arena through the glass bottoms of the testing bowls by a video camera located below the shielded enclosure. The body axis directions of each of the turtles were measured on 36 frames (0.9 frames per minute, 40 minutes). All measurements were done following a double blind procedure. Video recordings were given arbitrary numbers using a random sequence by a person not involved in the experimental design or behavioral measurements (M.P.). A single observer (L.L.) then measured directions for each of the videos in random order without knowing the identity of the turtles, treatment, or test day.

The posture of each animal was recorded and categorized for each frame using the categories “neutral posture”, “probably moving—direction not measureable”, or “crawling up against the wall”, measurements in the latter two groups were excluded from the analysis. Overall, 3.5% of the measurements were excluded (409 out of 11664). All measures were obtained using a double blind protocol from a single person (described above), to avoid observer biases.

### The magnetic field manipulations and radio frequency interference

The animals were tested in four different alignments of an earth-strength field (51.24 ± 0.06 μT, inclination angle 64.3° ± 0.6°), with magnetic north aligned to topographic north, east, south or west. The magnetic fields were generated by a pair of horizontally aligned, double-wrapped Rubens coils [73, 74]. The magnetic field components generated by the two coils were controlled by reversing the direction of current flow through one of the two wraps [5, 74]. For the vertical field condition, the horizontal field component was cancelled resulting in field strength of 46.78 μT (the residual of the horizontal component was 0.7 μT, towards 348°).

The radio frequency field was generated using a signal generator (Agilent, model 33250a), amplifier (Amplifier Research Associates, model 10A250), and an antenna consisting of a single, horizontal loop of wire surrounding the testing apparatus. The angle between the RF field and the magnetic field (26 ± 2°) was the same in all four horizontal alignments of the earth-strength magnetic field, and similar to that shown to disorient magnetic compass orientation in an earlier study of migratory birds [61]. The AC field was set to approximate the Larmor frequency for the ambient magnetic field strength (1.430 MHz). The intensity was measured at the location of each testing chamber and varied from 30 to 52nT, depending on the location. Although variation in RF conditions were recorded across the testing apparatus (higher RF intensities occurred around the periphery of the cluster of testing chambers, i.e., in chambers located closer to the radio antenna), each turtle remained in the same testing chamber and therefore, the same absolute position throughout the experiment, and thus experienced a constant RF field intensity in the treatment conditions in which it was exposed to the RF stimulus.

Testing in four symmetrical magnetic field alignments (magnetic north aligned to each of the cardinal compass directions), both with and without RF exposure, made it possible to partition the directional responses of individual turtles into magnetic and non-magnetic (topographic) components [62]. For example, to determine the topographic component of one of the turtles in the absence of RF exposure (i.e., ‘RF-off’), the directional responses from the four testing conditions were pooled without taking into account the magnetic field alignments. The vector sum of all individual responses from the four testing conditions pooled in this way provides a measure of the turtle’s response to any consistent non-magnetic (i.e., topographic) cues present in the testing chamber. To determine the magnetic component of the turtle’s response, the individual responses obtained in the same four testing conditions were pooled after first being rotated so that the directions of magnetic north coincided. The vector sum of all bearings of one individual pooled in this way provides a measure of the turtle’s response to the alignment of the static magnetic field. Similar analyses of the directional responses in the four field conditions in which the turtle was exposed to the RF field was then carried out to determine the topographic and magnetic components of response when exposed to the Larmor frequency field (‘RF on’).

The magnetic and topographic responses of the individuals were then summed for each of the conditions shown in S1 Table: 1) ‘RF off → RF off’, 2) ‘RF on → RF off’, 3) ‘RF on → RF on’, 4) ‘RF off → RF on’, 5) ‘RF off → vertical field (RF off)’, 6) ‘RF on vertical field (RF off)’.

All second order circular distributions of topographic and magnetic responses (each individual presented by a single vector) were analyzed with a second order Moore´s modified Rayleigh-test [75]. For comparisons of paired directional observations (‘RF off → RF off’ with ‘RF off → RF on’ and ‘RF on → RF off’ with ‘RF on → RF on’) the Moore’s paired sample test was used. For independent comparisons (‘RF off → RF off’ with ‘RF on → RF on’, ‘RF off → RF off’ with ‘RF on → RF off’, ‘RF off → RF on’ with ‘RF on → RF off’, ‘RF off → RF on’ with ‘RF on → RF on’) a Mardia’s two-sample comparison test was applied [76].

### Ethics Statement

Animal collections were permitted by the State of Virginia (Permit #: VA department of Game and Inland Fisheries #035981). The presented research was approved by the Institutional Animal Care and Use Committee of the Virginia Tech (#09-080-FIW, Amendment #2). Experiments were purely observational; the animals were released unharmed to their original habitat at the conclusion of the experiments.

## Supporting Information

S1 Dataset First order responses of all tested animals. (XLSX)

S2 Dataset Second order responses of all tested animals. (XLSX)

S1 Fig Alignment in vertical magnetic field. The results of the vertical field treatments (no RF was applied during this treatment). The results were indistinguishable from random for the days with (red outer circle) and without (black outer circle) RF acclimation. Moore’s modified Rayleigh test was used to test each distribution for significant unimodal alignment.
(PDF)

S2 Fig Topographic component of responses. The results combining the four magnetic field treatments (N, E, S, W), however, analyzed with regard to the topographic (geographic) north, showing that there was no topographic bias in the distribution. ‘RF off’ acclimated animals are shown with a black outer circle, ‘RF on’ acclimated animals with a red outer circle. Moore’s modified Rayleigh test was used to test each distribution for significant unimodal alignment. Dependent data (alignment of same individuals in the two treatments ‘RF off’ and ‘RF on’) were tested for significant differences using the Moore’s paired sample test. Independent data were tested for significant differences using the Mardia’s two-sample test.
(PDF)

S1 Table Testing schedule. (DOCX)

S2 Table GPS locations of collections of gravid females. (DOCX)

## References

[1] Lohmann KJ. Magnetic orientation by hatchling loggerhead sea turtles (*Caretta caretta*). J Exp Biol. 1991;155(1):37–49. 2016575 10.1242/jeb.155.1.37

[2] Lohmann KJ, Lohmann CMF. A light-independent magnetic compass in the leatherback sea turtle. Biol Bull. 1993;185(1):149–51. 29300602 10.2307/1542138

[3] Mathis A, Moore FR. Geomagnetism and the homeward orientation of the box turtle, *Terrapene carolina* . Ethology. 1988;78(4):265–74.

[4] Wiltschko W, Wiltschko R. Magnetic orientation and magnetoreception in birds and other animals. J Comp Physiol A Sens Neural Behav Physiol. 2005;191(8):675–93. 10.1007/s00359-005-0627-7 15886990

[5] Kirschvink JL, Winklhofer M, Walker MM. Biophysics of magnetic orientation: strengthening the interface between theory and experimental design. J R Soc Interface. 2010;7 Suppl 2:S179–S91. 10.1098/rsif.2009.0491.focus 20071390 PMC2843999

[6] Kirschvink JL, Walker MM, Diebel CE. Magnetite-based magnetoreception. Curr Opin Neurobiol. 2001;11(4):462–7. 11502393 10.1016/s0959-4388(00)00235-x

[7] Phillips JB, Jorge PE, Muheim R. Light-dependent magnetic compass orientation in amphibians and insects: candidate receptors and candidate molecular mechanisms. J R Soc Interface. 2010;7 Suppl 2:S241–56. 10.1098/rsif.2009.0459.focus 20124357 PMC2843995

[8] Winklhofer M, Kirschvink JL. A quantitative assessment of torque-transducer models for magnetoreception. J R Soc Interface. 2010;7(Suppl 2):S273–S89. 10.1098/rsif.2009.0435.focus 20086054 PMC2843997

[9] Walker MM. A model for encoding of magnetic field intensity by magnetite-based magnetoreceptor cells. J Theor Biol. 2008;250(1):85–91. 18028964 10.1016/j.jtbi.2007.09.030

[10] Ritz T, Ahmad M, Mouritsen H, Wiltschko R, Wiltschko W. Photoreceptor-based magnetoreception: optimal design of receptor molecules, cells, and neuronal processing. J R Soc Interface. 2010;7 Suppl 2:S135–S46. 10.1098/rsif.2009.0456.focus 20129953 PMC2843994

[11] Ritz T. Quantum effects in biology: Bird navigation. Procedia Chem. 2011;3(1):262–75.

[12] Stoneham AM, Gauger EM, Porfyrakis K, Benjamin SC, Lovett BW. A New Type of Radical-Pair-Based Model for Magnetoreception. Biophys J. 2012;102(5):961–8. 10.1016/j.bpj.2012.01.007 22404918 PMC3296028

[13] Lau JCS, Rodgers CT, Hore PJ. Compass magnetoreception in birds arising from photo-induced radical pairs in rotationally disordered cryptochromes. J R Soc Interface. 2012;9:3329–37. 10.1098/rsif.2012.0374 22977104 PMC3481564

[14] Ritz T, Adem S, Schulten K. A model for photoreceptor-based magnetoreception in birds. Biophys J. 2000;78(2):707–18. 10653784 10.1016/S0006-3495(00)76629-X PMC1300674

[15] Wiltschko R, Stapput K, Ritz T, Thalau P, Wiltschko W. Magnetoreception in birds: different physical processes for two types of directional responses. HFSP J. 2007;1(1):41–8. 10.2976/1.2714294/10.2976/1 19404459 PMC2645559

[16] Marhold S, Wiltschko W, Burda H. A magnetic polarity compass for direction finding in a subterranean mammal. Naturwissenschaften. 1997;84(9):421–3.

[17] Wang Y, Pan Y, Parsons S, Walker M, Zhang S. Bats respond to polarity of a magnetic field. Proceedings of the Royal Society B: Biological Sciences. 2007;274(1627):2901–5. 17848365 10.1098/rspb.2007.0904 PMC2288691

[18] Phillips JB, Muheim R, Jorge PE. A behavioral perspective on the biophysics of the light-dependent magnetic compass: a link between directional and spatial perception? J Exp Biol. 2010;213(19):3247–55. 10.1242/jeb.020792 20833916

[19] Wiltschko R, Stapput K, Bischof HJ, Wiltschko W. Light-dependent magnetoreception in birds: increasing intensity of monochromatic light changes the nature of the response. Front Zool. 2007;4:5 17302975 10.1186/1742-9994-4-5 PMC1810254

[20] Wiltschko R, Munro U, Ford H, Stapput K, Wiltschko W. Light-dependent magnetoreception: orientation behaviour of migratory birds under dim red light. J Exp Biol. 2008;211(Pt 20):3344–50. 10.1242/jeb.020313 18840669

[21] Keary N, Ruploh T, Voss J, Thalau P, Wiltschko R, Wiltschko W, et al Oscillating magnetic field disrupts magnetic orientation in Zebra finches, *Taeniopygia guttata* . Front Zool. 2009;6:25 10.1186/1742-9994-6-25 19852792 PMC2774300

[22] Phillips JB. Two magnetoreception pathways in a migratory salamander. Science. 1986;233(4765):765–7. 3738508 10.1126/science.3738508

[23] Phillips JB, Borland SC. Behavioural evidence for use of a light-dependent magnetoreception mechanism by a vertebrate. Nature. 1992;359:142–4.

[24] Phillips JB, Borland SC. Wavelength specific effects of light on magnetic compass orientation of the eastern red-spotted newt *Notophthalmus viridescens* . Ethol Ecol Evol. 1992;4(1):33–42.

[25] Phillips JB, Adler K, Borland SC. True navigation by an amphibian. Anim Behav. 1995;50(3):855–8.

[26] Munro U, Munro JA, Phillips JB, Wiltschko R, Wiltschko W. Evidence for a magnetite-based navigational 'map' in birds. Naturwissenschaften. 1997;84(1):26–8.

[27] Diego-Rasilla FJ, Luengo RM, Phillips JB. Use of a light-dependent magnetic compass for y-axis orientation in European common frog (*Rana temporaria*) tadpoles. J Comp Physiol A Sens Neural Behav Physiol. 2013;199:1–10. 10.1007/s00359-013-0811-0 23525820

[28] Vácha M, Soukopova H. Magnetic orientation in the mealworm beetle *Tenebrio* and the effect of light. J Exp Biol. 2004;207(Pt 7):1241–8. 14978064 10.1242/jeb.00874

[29] Vácha M, Puzova T, Drstkova D. Effect of light wavelength spectrum on magnetic compass orientation in *Tenebrio molitor* . J Comp Physiol A Sens Neural Behav Physiol. 2008;194(10):853–9. 10.1007/s00359-008-0356-9 18696079

[30] Wiltschko R, Stapput K, Thalau P, Wiltschko W. Directional orientation of birds by the magnetic field under different light conditions. J R Soc Interface. 2009;7(Suppl 2):S163—S77. 10.1098/rsif.2009.0367.focus 19864263 PMC2843996

[31] Phillips JB, Sayeed O. Wavelength-dependent effects of light on magnetic compass orientation in *Drosophila melanogaster* . J Comp Physiol A Sens Neural Behav Physiol. 1993;172(3):303–8. 10.1007/BF00216612 8510055

[32] Wiltschko W, Wiltschko R. Magnetic compass of European robins. Science. 1972;176(4030):62–4. 17784420 10.1126/science.176.4030.62

[33] Deutschlander ME, Phillips JB, Borland SC. The case for light-dependent magnetic orientation in animals. J Exp Biol. 1999;202 (Pt 8):891–908. 10085262 10.1242/jeb.202.8.891

[34] Painter MS, Dommer DH, Altizer WW, Muheim R, Phillips JB. Spontaneous magnetic orientation in larval *Drosophila* shares properties with learned magnetic compass responses in adult flies and mice. J Exp Biol. 2013;216:1307–16. 10.1242/jeb.077404 23239891

[35] Nießner C, Denzau S, Gross JC, Peichl L, Bischof HJ, Fleissner G, et al Avian Ultraviolet/Violet cones identified as probable magnetoreceptors. PLOS ONE. 2011;6(5): e20091 10.1371/journal.pone.0020091 21647441 PMC3102070

[36] Phillips JB, Borland S. Use of a specialized magnetoreception system for homing by the eastern red-spotted newt *Notophthalmus viridescens* . J Exp Biol. 1994;188(1):275–91. 9317797 10.1242/jeb.188.1.275

[37] Wiltschko R, Thalau P, Gehring D, Nießner C, Ritz T, Wiltschko W. Magnetoreception in birds: the effect of radio-frequency fields. J R Soc Interface. 2015;12(103). 10.1098/rsif.2014.1103 PMC4305412 25540238

[38] Wiltschko W, Munro U, Ford H, Wiltschko R. Bird navigation: what type of information does the magnetite-based receptor provide? Proc R Soc Lond B. 2006;273:2815–20. 17015316 10.1098/rspb.2006.3651 PMC1664630

[39] Light P, Salmon M, Lohmann K. Geomagnetic orientation of loggerhead sea turtles: evidence for an inclination compass. J Exp Biol. 1993;182(1):1–10.

[40] Henbest KB, Kukura P, Rodgers CT, Hore PJ, Timmel CR. Radio frequency magnetic field effects on a radical recombination reaction: a diagnostic test for the radical pair mechanism. J Am Chem Soc. 2004;126(26):8102–3. 15225036 10.1021/ja048220q

[41] Wiltschko W, Wiltschko R, Ritz T. The mechanism of the avian magnetic compass. Procedia Chem. 2011;3(1):276–84.

[42] Engels S, Schneider N-L, Lefeldt N, Hein CM, Zapka M, Michalik A, et al Anthropogenic electromagnetic noise disrupts magnetic compass orientation in a migratory bird. Nature. 2014;509:353–6. 10.1038/nature13290 24805233

[43] Thalau P, Ritz T, Burda H, Wegner RE, Wiltschko R. The magnetic compass mechanisms of birds and rodents are based on different physical principles. J R Soc Interface. 2006;3(9):583–7. 16849254 10.1098/rsif.2006.0130 PMC1664646

[44] Becker G. Reaktion von Insekten auf Magnetfelder, elektrische Felder und atmospherics. Zeitschrift für angewandte Entomologie. 1964;54(14):75–88. 10.1107/S0108767309007235 19349661

[45] Begall S, Červený J, Neef J, Vojtěch O, Burda H. Magnetic alignment in grazing and resting cattle and deer. Proc Natl Acad Sci. 2008;105(36):13451–5. 10.1073/pnas.0803650105 18725629 PMC2533210

[46] Červený J, Begall S, Koubek P, Nováková P, Burda H. Directional preference may enhance hunting accuracy in foraging foxes. Biol Lett. 2011;7(3):355–7. 10.1098/rsbl.2010.1145 21227977 PMC3097881

[47] Hart V, Kušta T, Němec P, Bláhová V, Ježek M, Nováková P, et al Magnetic Alignment in Carps: Evidence from the Czech Christmas Fish Market. PLOS ONE. 2012;7(12):e51100 10.1371/journal.pone.0051100 23227241 PMC3515494

[48] Burda H, Begall S, Červený J, Neef J, Němec P. Extremely low-frequency electromagnetic fields disrupt magnetic alignment of ruminants. Proc Natl Acad Sci. 2009;106(14):5708–13. 10.1073/pnas.0811194106 19299504 PMC2667019

[49] Begall S, Burda H, Červený J, Gerter O, Neef-Weisse J, Němec P. Further support for the alignment of cattle along magnetic field lines: reply to Hert et al J Comp Physiol A Sens Neural Behav Physiol. 2011:1–7. 10.1007/s00359-011-0674-1 PMC3221857 22028177

[50] Begall S, Malkemper EP, Červený J, Němec P, Burda H. Magnetic alignment in mammals and other animals. Mamm Biol. 2013;78:10–20.

[51] Phillips JB, Youmans PW, Muheim R, Sloan KA, Landler L, Painter MS, et al Rapid Learning of Magnetic Compass Direction by C57BL/6 Mice in a 4-Armed ‘Plus’ Water Maze. PLOS ONE. 2013;8(8):e73112 10.1371/journal.pone.0073112 24023673 PMC3758273

[52] Phillips JB, Borland SC, Freake MJ, Brassart J, Kirschvink JL. 'Fixed-axis' magnetic orientation by an amphibian: non-shoreward-directed compass orientation, misdirected homing or positioning a magnetite-based map detector in a consistent alignment relative to the magnetic field? J Exp Biol. 2002;205:3903–14. 12432012 10.1242/jeb.205.24.3903

[53] Liedvogel M, Mouritsen H. Cryptochromes—a potential magnetoreceptor: what do we know and what do we want to know? J R Soc Interface. 2010;7(Suppl 2):S147–S62. 10.1098/rsif.2009.0411.focus 19906675 PMC2844001

[54] Gegear RJ, Casselman A, Waddell S, Reppert SM. Cryptochrome mediates light-dependent magnetosensitivity in *Drosophila* . Nature. 2008;454(7207):1014–8. 10.1038/nature07183 18641630 PMC2559964

[55] Bailey MJ, Chong NW, Xiong J, Cassone VM. Chickens’ Cry2: molecular analysis of an avian cryptochrome in retinal and pineal photoreceptors. FEBS letters. 2002;513(2):169–74. 11904144 10.1016/s0014-5793(02)02276-7

[56] Nießner C, Denzau S, Stapput K, Ahmad M, Peichl L, Wiltschko W, et al Magnetoreception: activated cryptochrome 1a concurs with magnetic orientation in birds. J R Soc Interface. 2013;10(88):20130638 10.1098/rsif.2013.0638 23966619 PMC3785833

[57] Mazzotta G, Rossi A, Leonardi E, Mason M, Bertolucci C, Caccin L, et al Fly cryptochrome and the visual system. Proc Natl Acad Sci. 2013;110(15):6163–8. 10.1073/pnas.1212317110 23536301 PMC3625353

[58] Solov´yov IA, Hore PJ, Ritz T, Schulten K. A chemical compass for bird navigation Quantum Effects in Biology. New York City: Cambridge University Press; 2011.

[59] Maeda K, Henbest KB, Cintolesi F, Kuprov I, Rodgers CT, Liddell PA, et al Chemical compass model of avian magnetoreception. Nature. 2008;453(7193):387–90. 10.1038/nature06834 18449197

[60] Dodson CA, Hore PJ, Wallace MI. A radical sense of direction: signalling and mechanism in cryptochrome magnetoreception. Trends Biochem Sci. 2013;429(6988):177–80. 10.1016/j.tibs.2013.07.002 23938034

[61] Ritz T, Thalau P, Phillips JB, Wiltschko R, Wiltschko W. Resonance effects indicate a radical-pair mechanism for avian magnetic compass. Nature. 2004;38(9):435–46. 10.1038/nature02534 15141211

[62] Muheim R, Edgar NM, Sloan KA, Phillips JB. Magnetic compass orientation in C57BL/6J mice. Learn Behav. 2006;34(4):366–73. 17330527 10.3758/bf03193201

[63] Phillips JB. Magnetic compass orientation in the Eastern red-spotted newt (*Notophthalmus viridescens*). J Comp Physiol A Sens Neural Behav Physiol. 1986;158(1):103–9. 10.1007/BF00614524 3723427

[64] Muheim R, Phillips JB, Åkesson S. Polarized light cues underlie compass calibration in migratory songbirds. Science. 2006;313(5788):837–9. 16902138 10.1126/science.1129709

[65] Collett TS, Baron J. Biological compasses and the coordinate frame of landmark memories in honeybees. Nature. 1994;368(6467):137–40.

[66] Collett T. Landmark learning and guidance in insects. Philos Trans R Soc Lond B Biol Sci. 1992:295–303. 1354368

[67] Cartwright B, Collett TS. Landmark learning in bees. Journal of comparative physiology. 1983;151(4):521–43. 10.1007/BF01324825 3735168

[68] Ritz T, Wiltschko R, Hore PJ, Rodgers CT, Stapput K, Thalau P, et al Magnetic compass of birds is based on a molecule with optimal directional sensitivity. Biophys J. 2009;96:3451–7. 10.1016/j.bpj.2008.11.072 19383488 PMC2718301

[69] Xu B-M, Zou J, Li H, Li J-G, Shao B. Effect of radio frequency fields on the radical pair magnetoreception model. Phys Rev E. 2014;90(4):042711. 10.1103/PhysRevE.90.042711 25375527

[70] Vácha M, Puzova T, Kvicalova M. Radio frequency magnetic fields disrupt magnetoreception in American cockroach. J Exp Biol. 2009;212(21):3473–77. 10.1242/jeb.028670 19837889

[71] Hopkins BC, Hepner MJ, Hopkins WA. Non-destructive techniques for biomonitoring of spatial, temporal, and demographic patterns of mercury bioaccumulation and maternal transfer in turtles. Environ Pollut. 2013;177:164–70. 10.1016/j.envpol.2013.02.018 23500054

[72] Hopkins B, Willson JD, Hopkins W. Mercury exposure is associated with negative effects on turtle reproduction. Environ Sci Technol. 2013;47:2416–22. 10.1021/es304261s 23360167

[73] Rubens SM. Cube-surface coil for producing a uniform magnetic field. Rev Sci Instrum. 1945;16:243.

[74] Kirschvink JL. Uniform magnetic fields and double wrapped coil systems: Improved techniques for the design of bioelectromagnetic experiments. Bioelectromagnetics. 1992;13(5):401–11. 1445421 10.1002/bem.2250130507

[75] Moore BR. A modification of the Rayleigh test for vector data. Biometrika. 1980;67(1):175–80.

[76] Mardia K. A non-parametric test for the bivariate two-sample location problem. J R Stat Soc Series B Stat Methodol. 1967;29(2):320–42.

