# Supplementary material for: Spontaneous Magnetic Alignment by Yearling Snapping Turtles: Rapid Association of Radio Frequency Dependent Pattern of Magnetic Input with Novel Surroundings
Source: PLoS One. 2015 May 15;10(5):e0124728. doi: 10.1371/journal.pone.0124728 (PMC4433231; doi:10.1371/journal.pone.0124728)
Supplement: S1 Fig — The results of the vertical field treatments (no RF was applied during this treatment). The results were indistinguishable from random for the days with (red outer circle) and without (black outer circle) RF acclimation. Moore’s modified Rayleigh test was used to test each distribution for significant unimodal alignment. (PDF) [file pone.0124728.s003.pdf]

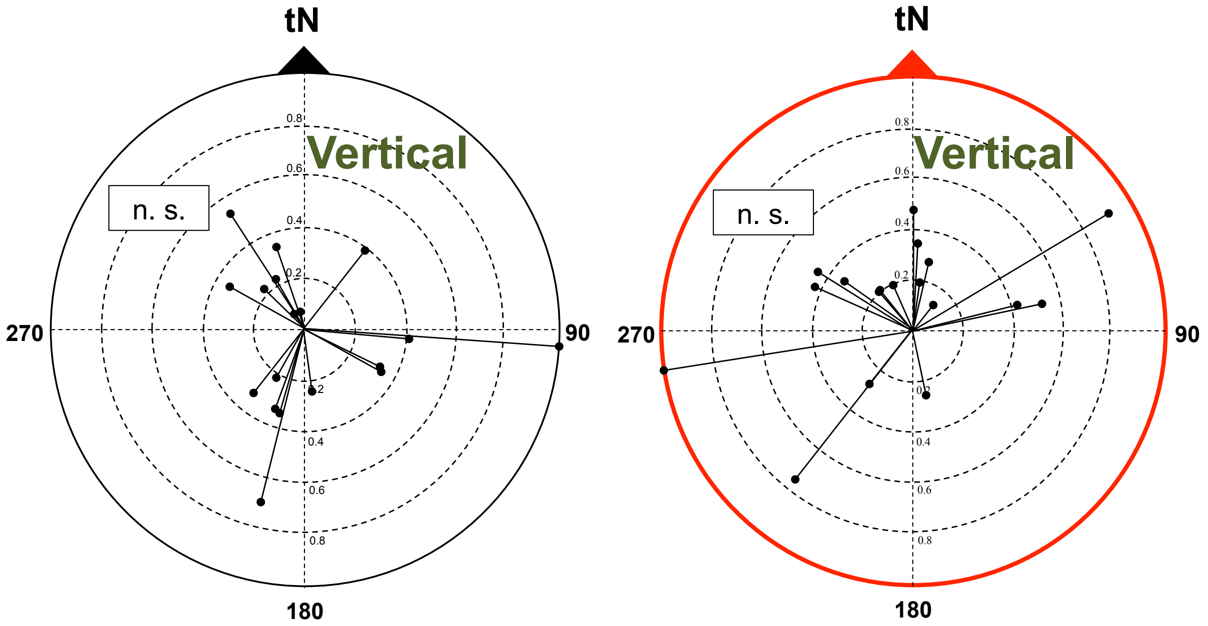

S1 Fig. Alignment in vertical magnetic field. The results of the vertical field treatments (no RF was applied during this treatment). The results show random orientation for the days with (red outer circle) and without (black outer circle) RF acclimation. Moore's modified Rayleigh test was used to test each distribution for significant unimodal alignment.
