# Supplementary material for: Spontaneous Magnetic Alignment by Yearling Snapping Turtles: Rapid Association of Radio Frequency Dependent Pattern of Magnetic Input with Novel Surroundings
Source: PLoS One. 2015 May 15;10(5):e0124728. doi: 10.1371/journal.pone.0124728 (PMC4433231; doi:10.1371/journal.pone.0124728)
Supplement: S2 Fig — The results combining the four magnetic field treatments (N, E, S, W), however, analyzed with regard to the topographic (geographic) north, showing that there was no topographic bias in the distribution. ‘RF off’ acclimated animals are shown with a black outer circle, ‘RF on’ acclimated animals with a red outer circle. Moore’s modified Rayleigh test was used to test each distribution for significant unimodal alignment. Dependent data (alignment of same individuals in the two treatments ‘RF off’ and ‘RF on’) were tested for significant differences using the Moore’s paired sample test. Independent data were tested for significant differences using the Mardia’s two-sample test. (PDF) [file pone.0124728.s004.pdf]

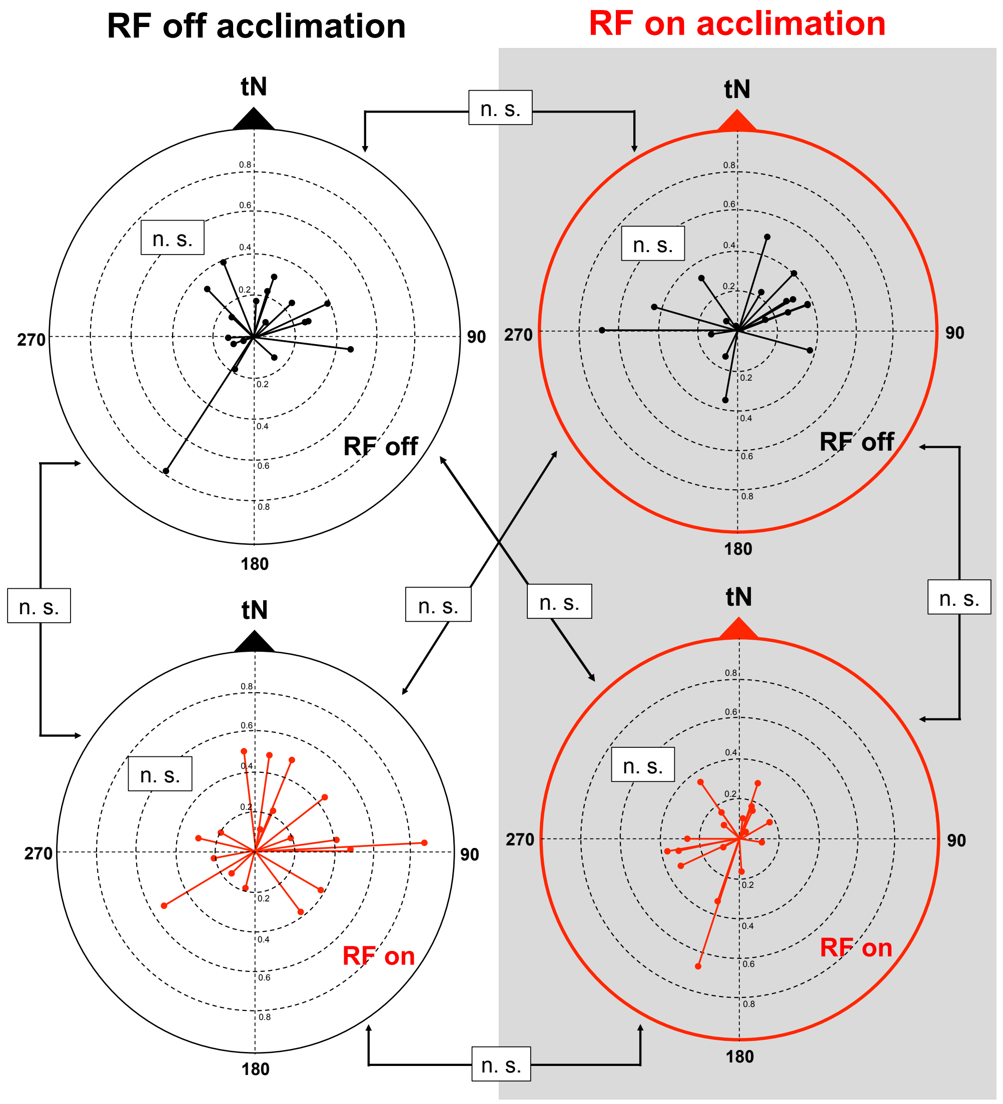

S2 Fig. Topographic component of responses. The results combing the 4 magnetic field treatments (N, E, S, W), however, analyzed with regard to the topographic (geographic) north, showing that there is no topographic bias in the data. RF off acclimated animals are shown with a black outer circle, RF on acclimated animals with a red outer circle. Moore's modified Rayleigh test was used to test each distribution for significant unimodal alignment. Dependent data (alignment of same individuals in the two treatments RF off and RF on) were tested for significant differences using the Moore's paired sample test. Independent data were tested for significant differences using the Mardia's two-sample test.
