# Supplementary material for: Spontaneous Magnetic Alignment by Yearling Snapping Turtles: Rapid Association of Radio Frequency Dependent Pattern of Magnetic Input with Novel Surroundings
Source: PLoS One. 2015 May 15;10(5):e0124728. doi: 10.1371/journal.pone.0124728 (PMC4433231; doi:10.1371/journal.pone.0124728)
Supplement: S1 Table — (DOCX) [file pone.0124728.s005.docx]

**S1 Table. Testing schedule.**

RF = radio frequency treatment, VF = vertical field, N = magnetic north aligned towards topographic north, E = magnetic north aligned towards topographic east, S = magnetic north aligned towards topographic south, W = magnetic north aligned towards topographic west.
