# Supplementary material for: Spontaneous Magnetic Alignment by Yearling Snapping Turtles: Rapid Association of Radio Frequency Dependent Pattern of Magnetic Input with Novel Surroundings
Source: PLoS One. 2015 May 15;10(5):e0124728. doi: 10.1371/journal.pone.0124728 (PMC4433231; doi:10.1371/journal.pone.0124728)
Supplement: S2 Table — (DOCX) [file pone.0124728.s006.docx]

**S2 Table. GPS locations of collections of gravid females.**

| **Female Turtle ID** | **Eastings** | **Northings** |
| --- | --- | --- |
| **CIR** | 0656581 | 4224322 |
| **COX** | 0681744 | 4212506 |
| **CIJ** | 0656216 | 4224150 |
| **CIN** | 0656449 | 4224321 |
| **CIT** | 0656456 | 4224325 |
| **CJO** | 0656201 | 4224136 |
| **CLU** | 0656470 | 4224325 |
| **CIL** | 0656449 | 4224321 |
| **CKN** | 0656216 | 4224150 |
| **BHR** | 0656418 | 4224311 |
| **BHQ** | 0656470 | 4224168 |
| **COS** | 0681922 | 4212594 |
